# Ozone control as a novel method to improve health-promoting bioactive compounds in red leaf lettuce (*Lactuca sativa* L.)

**DOI:** 10.3389/fpls.2022.1045239

**Published:** 2022-12-05

**Authors:** Jin-Hui Lee, Eiji Goto

**Affiliations:** ^1^ Graduate School of Horticulture, Chiba University, Chiba, Japan; ^2^ Plant Molecular Research Center, Chiba University, Chiba, Japan

**Keywords:** air pollution, antioxidant capacity, environmental stress, ozone detoxification and repair, ozone sensitivity, phytochemical, vertical farm

## Abstract

In this study, we determined the short-term effects of ozone exposure on the growth and accumulation of bioactive compounds in red lettuce leaves grown in a controlled environment plant factory with artificial light, also known as a vertical farm. During cultivation, twenty-day-old lettuce (*Lactuca sativa* L. var. Redfire) seedlings were exposed to 100 and 200 ppb of ozone concentrations for 72 h. To find out how plants react to ozone and light, complex treatments were done with light and ozone concentrations (100 ppb; 16 h and 200 ppb; 24 h). Ozone treatment with 100 ppb did not show any significant difference in shoot fresh weight compared to that of the control, but the plants exposed to the 200 ppb treatment showed a significant reduction in fresh weight by 1.3 fold compared to the control. The expression of most genes in lettuce plants exposed to 100 and 200 ppb of ozone increased rapidly after 0.5 h and showed a decreasing trend after reaching a peak. Even when exposed to a uniform ozone concentration, the pattern of accumulating bioactive compounds such as total phenolics, antioxidant capacity and total flavonoids varied based on leaf age. At a concentration of 200 ppb, a greater accumulation was found in the third (older) leaf than in the fourth leaf (younger). The anthocyanin of lettuce plants subjected to 100 and 200 ppb concentrations increased continuously for 48 h. Our results suggest that ozone control is a novel method that can effectively increase the accumulation of bioactive compounds in lettuce in a plant factory.

## Introduction

Phytochemicals are naturally occurring bioactive compounds found in plants. Numerous forms of antioxidant phytochemicals in plants have been linked to the prevention of chronic diseases including cancer and cardiovascular disease (Rajashekar et al., 2009). The emergence of several diseases are linked to oxidative stress induced by reactive oxygen species (ROS). Antioxidant phytochemicals are known for their neutralization effect on free radicals with high oxidation potential. Consequently, the popularity of diets containing antioxidant phytochemicals continues to rise (Tomasetti et al., 2017).

Tropospheric-level ozone (O_3_) is a major air contaminant. The oxidative damage caused by ozone to the physiological and biochemical processes of plants is a serious global concern (Booker et al., 2012). According to a previous study, exposure to ozone concentrations of 80 ppb for 6.6 h resulted in decreased lung function and an increased inflammatory response in humans (Brown, 2009). Ozone induces substantial alterations in plant as well as human metabolism due to its strong oxidizing capacity (Lorenzini and Nali, 2018). Consequently, it affects plant growth, quality, and yield (Overmyer et al., 2003; Kangasjärvi et al., 2005). When plants are exposed to ozone, they trigger the expression of genes and subsequently accumulate ROS-scavengers and other metabolites as part of their antioxidant defense mechanism (Booker et al., 2012). In response to oxidative stress caused by ground-level ozone concentrations, an increase in antioxidant-related enzyme activity is observed in plants (Shao et al., 2007; Burkart et al., 2013; Sarkar et al., 2015). An increase in phenylpropanoid metabolites and the formation of various phenolic compounds have been reported in plants exposed to ozone. This further stimulates plant cell metabolism to restore and maintain cellular structure (Dizengremel, 2001; Andersen, 2003).

Since ROS are generated when plants are exposed to oxidative stress, especially after ozone uptake, the antioxidant capacity of the leaf apoplast is crucial for determining ozone tolerance (Podila et al., 2001). The response of plants to ozone varies depending on ozone concentration, duration of exposure, plant developmental stage, and stomatal conductance (De Temmerman et al., 2002). In addition to ascorbic acid and glutathione, various substances including phenols help scavenge ROS (Podila et al., 2001; De Temmerman et al., 2002). However, unlike other stress inducing substances, ozone acts as an elicitor and decomposes into oxygen, leaving no toxic residue behind (Pellegrini et al., 2018). In atmospheric systems, UV and ozone are closely correlated and have been reported to have similar effects on the mRNA accumulation of antioxidant genes in *Nicotiana plumbaginifolia* L. plants (Willekens et al., 1994). Unlike UV radiation, which is absorbed only on the leaf surface, ozone is easily absorbed by plant organs through the stomata and cuticular layers. After ozone penetrates the leaf tissue, O3 immediately interacts with extracellular antioxidants, which appear to stimulate ascorbate synthesis and/or its transport between compartments (Heath, 2008). Therefore, it could be said that ozone boosts the accumulation of bioactive antioxidants in plants (Sandermann et al., 1998).

A plant factory with artificial light (PFAL), also called a vertical farm, is an advanced plant cultivation system which is used for the production of commercial leafy vegetables such as leaf lettuce (Zhang et al., 2018; He et al., 2021). The system is airtight without windows and completely controls such as light, temperature, humidity, as well as CO_2_ and O_2_ gases. To date, many research on the effects of light, air, and root temperatures, humidity, and CO_2_ concentration on dry mass production in lettuce and other leafy vegetables have been carried out in PFAL systems (Choi et al., 2000; Zhang et al., 2018; Carotti et al., 2021; He et al., 2021). As for the enhancement of health-promoting bioactive compounds in the plants, a variety of environmental parameters have been proposed as preharvest treatments which were effective in increasing the value of indoor farming produce (Lee et al., 2021). Ozone concentration can be controlled in a PFAL using a simple control unit with an ozone generator and a sensor. Compared to other chemical substances, the use of ozone is advantageous as it does not leave a residue; once generated, ozone is easily decomposed to O_2_ and H_2_O (Pellegrini et al., 2018). In addition, the plant factory has the advantage of being able to control the photoperiod because it uses artificial light. According to the results of previous studies, growth, net photosynthesis, and chlorophyll content were significantly decreased in unshaded-treated hybrid poplar (*Populus tristis* Fisch. × *P. balsamifera* L. cv. Tristis) compared to shaded-treated plants when combined treatment with light treatment (unshaded and shaded) and ozone was applied (Volin et al., 1993). Ozone induces stomata closure in leaves, which reduces CO_2_ absorption and ultimately increases sensitivity to photoinhibition in ozonated plants (Fredericksen et al., 1996; Topa et al., 2001). This means that ozone and the light environment can exhibit strong interactions, and light conditions can cause further damage to plants exposed to ozone stress (Hurry et al., 1992; Guidi et al., 2000). Therefore, if light and ozone are treated for 24 hours without a dark period, it is possible to stimulate the antioxidant system in a shorter time than with photoperiodic treatment. If ozone induces the production of health-promoting bioactive compounds in plants, it can very well be incorporated in a preharvest treatment plan in a PFAL for value-added plant production.

Plant defense mechanisms against ozone vary based on factors, such as the leaf growth stage (leaf age) and thickness, plant species, and ozone exposure dynamics. In addition, environmental conditions affect the morphological and physiological response of plants to ozone (Guderian, 1985). For example, woody plants upon exposure to ozone have shown differential changes in antioxidant levels at different stages of plant development or have shown changes at the same stage in a given day (Schupp and Rennenberg, 1988; Hausladen et al., 1990; Wieser et al., 2002; Nogués et al., 2008). Because it is difficult to expose large trees to ozone experimentally, studies have been conducted on woody plants mainly to determine the effect of age (developmental stages) on the response of trees to ozone. But only a few studies have shown that ozone has an effect on the stages of growth in herbaceous plants such as leafy vegetables. In the case of leafy vegetables such as Brassica (mustard, oilseed rape), there have been many studies related to ozone response based on crop or cultivars (Abedi and Pakniyat, 2010; Singh et al., 2011). When growing lettuce in a PFAL, it is important to monitor the ozone response as an elicitor because lettuce has a high economic and commercial value. Calatayud and Barreno (2004) confirmed that two lettuce varieties exhibited different chlorophyll a fluorescence reactions, photosynthetic pigmentations, and lipid peroxidation reactions when exposed to ozone. In addition, changes in photosynthetic CO_2_ exchange, chlorophyll a fluorescence, and yield of lettuce exposed to ozone at different growth stages have also been reported (Calatayud et al., 2002; Goumenaki and Barnes, 2009). Apart from photosynthetic parameters and yield/growth responses (Temple et al., 1986; Goumenaki et al., 2007; Kleiber et al., 2017), biochemical parameters such as the accumulation of antioxidant enzymes have been identified upon exposure to ozone before harvest (Calatayud et al., 2002). To the best of our knowledge, there is no information on age-dependent defense responses of lettuce plants exposed to various ozone concentrations.

This study aims to determine the effect of ozone concentrations on the accumulation of secondary metabolites in red leaf lettuce plants grown in a plant factory-like system. The objectives of this study were to determine: (1) changes in secondary metabolite pathways upon ozone exposure monitored throughout the day before harvest, (2) effects of short-term ozone exposure on the levels of antioxidant bioactive compounds, and (3) effects of photoperiod and ozone concentrations on the accumulation of bioactive compounds.

## Materials and methods

### Plant materials and environmental conditions

Red leaf lettuce (*Lactuca sativa* L. var. Redfire) seeds were placed in a wet paper towel (Kimtowel, Nippon Paper Crecia Co. Ltd., Tokyo, Japan) in a Petri plate, wrapped using a plastic wrap. The following day, germinated seeds were moved to a urethane sponge for seedling growth. Distilled water was supplied until 6 days after sowing (DAS). The seedlings were then transferred to a hydroponic system with an air pump and subsequently cultivated until 20 DAS. Thirty-two plants were grown per tray with a deep flow technique hydroponic system, and three trays were grown per experiment. Lettuce plants were grown in the plant factory until 20 DAS, after which a uniform plant was selected and transferred to the ozone chamber. One-fourth concentration of Otsuka A (OAT house A treatment; OAT Agrio Co. Ltd., Tokyo, Japan) nutrient recipe solution was supplied (EC: 1.0 ds m^-1^ and pH: 6.5). The environmental conditions for growing red leaf lettuce was set as follows: white LED lamps (LDL40S-N/19/21; Panasonic Corp., Osaka, Japan), at 200 μmol m^-2^ s^-1^ photosynthetic photon flux density (PPFD), 16 h light, 25°C/20°C (day/night) air temperature, 70% relative humidity (RH), and 1000 μmol mol^-1^ CO_2_ concentration. PPFD was measured at 12 points per tray, with a measurement range of 200 ± 10 μmol m^-2^ s^-1^. The PPFD of the white LED light was adjusted to be 200 PPFD on average.

#### Ozone treatment

**Figure 1:**
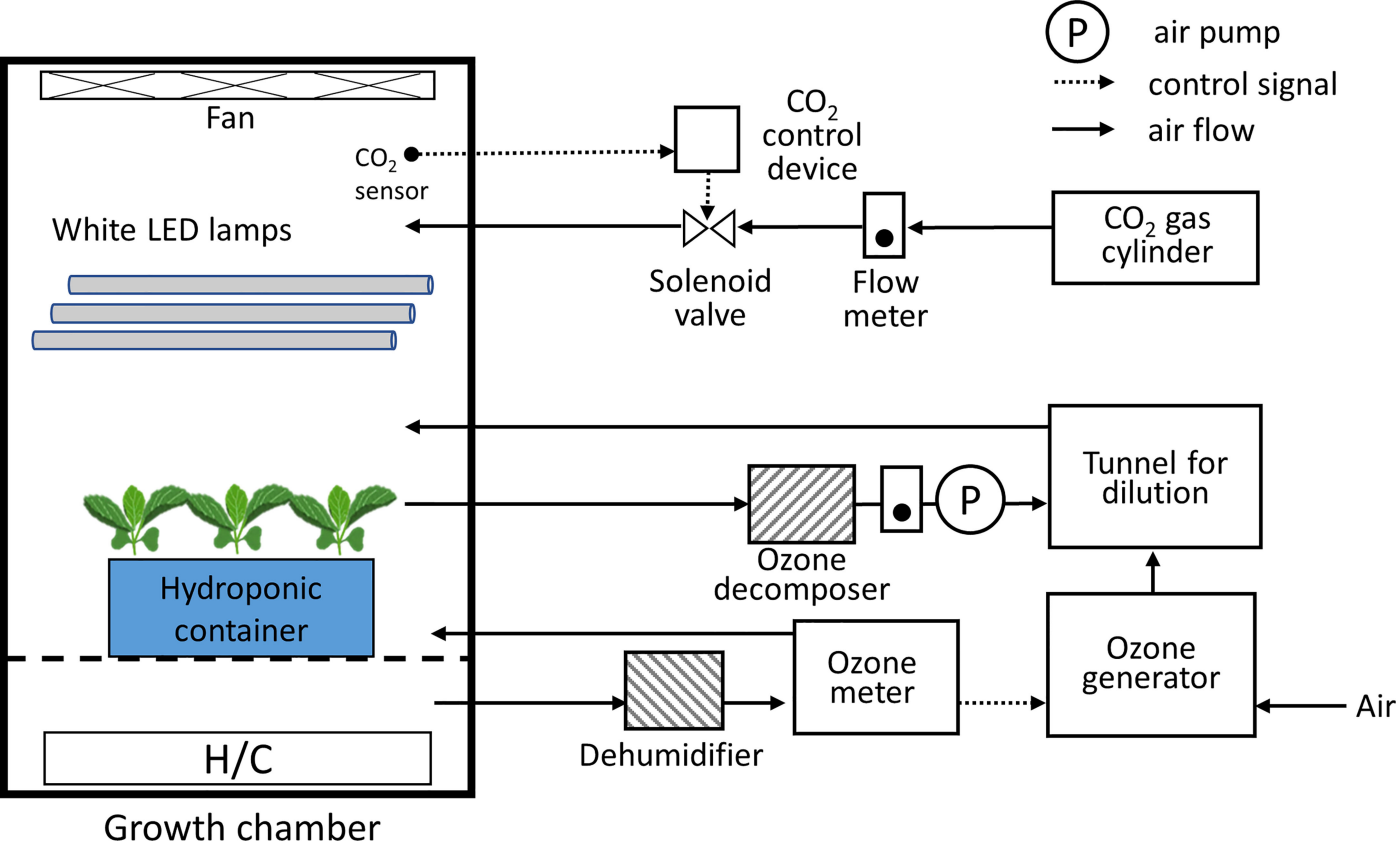
Schematic diagram of the growth chamber with an ozone concentration control equipment. The light period, photosynthetic photon flux density, CO_2_ concentration, O_3_ concentration, air temperature, and relative humidity in the chamber can be controlled.

The 20-day-old seedlings (20 DAS; about four true leaves appeared) were subjected to 100 and 200 ppb of ozone treatment and non-ozonated treatment (control). The environment of the ozone chamber remained the same as the plant factory’s cultivation area (200 PPFD, 25°C/20°C (day/night) air temperature, 70% RH, and 1000 μmol mol^-1^ CO_2_). Ozone treatment was given to the seedlings for 72 h. **Figure 1** and **Supplementary Figure S1** show the schematic diagram of the ozone chamber and the ozone treatment given in this experiment. Ozone concentration was controlled using the light ozone generator (WOR1040-Z1; Ushio Inc., Tokyo, Japan) and an ozone monitor (EG-700EIII; Ebara Jitsugyo Co. Ltd., Tokyo, Japan) in a growth chamber with white LEDs (FLI-2010H-LED; Tokyo Rikakikai Co. Ltd., Tokyo, Japan). Two experiments were conducted to confirm the effect of light period and ozone concentration (**Table 1**). In the case of 100 ppb, the light period was set to 16 h, the same as in the current cultivation environment. To exclude plant reactions owing to ozone absorption during the light and dark periods, the following ozone experiment at a concentration of 200 ppb was conducted using continuous light conditions. The control plants were also grown in the same model chamber as the ozone chamber of which ozone concentration was controlled zero. At each time point, both ozonated plants and control plants were taken at the same time for the analysis of bioactive compounds. Fresh weight of shoot and root were determined immediately before treatment and at 72 h of ozone treatment.

**Table 1:** Environmental conditions during ozone treatment.

	Seedling stage	Ozone treatment
Light period (h d^-1^) [100 ppb]	16	
Light period (h d^-1^) [200 ppb]	24	
PPFD* (μmol m^-2^ s^-1^)	100	200
Air temperature (°C)	25/20	
Relative humidity (%)	70	
CO_2_ concentration (μmol mol^-1^)	1000	

*Photosynthetic photon flux density on culture panels.

### Determination of total phenolics, antioxidant capacity, total flavonoids and anthocyanin

For the analysis of bioactive compounds, lettuce plants grown in ozone chambers or control chambers were randomly selected at every sampling point. Each leaf sample was collected at 0, 0.25, 0.5, 1, 2, 5, 8, 12, 16, 24, 48, and 72 hours after ozone exposure. In order to confirm the response according to the leaf age, the third and fourth leaves were sampled. Leaves were counted starting from bottom, excluding cotyledons. The collected leaf samples were stored in a deep freezer (-80°C) until further analysis. Total phenolic concentration and Trolox equivalent antioxidant capacity were analyzed using a slightly modified method by Miller and Rice-Evans (1996) and Ainsworth and Gillespie (2007). Approximately 50 mg of fresh leaf samples were used for the analyses. The remaining analytical procedures of the total phenolic concentration and antioxidant capacity were investigated as described (Lee et al., 2021).

Flavonoid concentration was determined using the method of Zhishen et al. (1999). One milliliter of 80% acetone was added to the powdered leaf sample and extracted at 16 Hz for 1 min using a MM400 ball mill (Retsch GmbH, Haan, Germany), and then sonicated at 30 Hz for 6 min. The extract was incubated overnight under darkness at 4°C. The assay mixture for the determination of total flavonoid concentration comprised distilled water, 5% NaNO_2_, leaf extract, 10% (w/v) AlCl_3_, 1M NaOH in a final mixture volume of 1.5 mL. The absorbance of flavonoid concentration was measured at 510 nm using a spectrophotometer (V-750; JASCO Corp., Tokyo, Japan) (mg catechin g^–1^ FW).

Anthocyanins concentration in red lettuce was measured with slight modifications to the analytical method of Mancinelli and Schwartz (1984). Leaves were used for the extraction of anthocyanin (50 mg fresh samples/400 mL of 1% (v/v) HCl in methanol). Briefly, the extracted samples were mixed with 200 μL distilled water and 500 μL chloroform and centrifuged for 2 min at 13,000 × g at 4°C. A 400 μL aliquot of the aqueous fraction of the sample was then mixed with 600 μL of 1% (v/v) HCl in methanol. The absorbance was read at 530 and 657 nm (A530-0.25 × A657) using a spectrophotometer (V-750; JASCO Corp., Tokyo, Japan). Results were expressed in ug C3G g^-1^ FW.

### Hydrogen peroxide (H_2_O_2_) determination

Hydrogen peroxide accumulation in red lettuce leaves exposed to two ozone concentrations was analyzed using the method of Velikova et al. (2000). The collected fresh leaf samples were ground and then extracted with 1 mL 0.1% (w/v) trichloroacetic acid. An aliquot (0.5 mL) of the supernatant of the extract was mixed with 10 mM potassium phosphate buffer (pH 7.0, 0.5 mL) and 1M KI (1 mL). The absorbance of hydrogen peroxide mixture was measured at 390 nm using a spectrophotometer (V-750; JASCO Corp., Tokyo, Japan). Results were expressed in H_2_O_2_ g^-1^ FW.

### Gene expression quantification

Total RNA was extracted from red leaf lettuce leaves (approximately 100–150 mg) using the RNeasy Plant Mini Kit (Qiagen N.V., Venlo, The Netherlands) after homogenization in liquid nitrogen. The fourth leaf from the bottom was sampled at 0, 0.25, 0.5, 1, 2, 5, 8, 12, 16, and 24 h of ozone exposure to examine time-dependent gene expression variations. The oligonucleotide primers used in this study were designed using data from the GenBank database (**Table 2**). The remaining analytical procedures (complementary DNA synthesis and PCR) were followed as described by Lee et al. (2021). The following mRNA transcripts were quantified: phenylalanine ammonia-lyase (*PAL*), cinnamic acid 4-hydroxylase (*C4H*), 4-coumaroyl-CoA ligase (*4CL*), chalcone synthase (*CHS*), chalcone isomerase (*CHI*), flavanone 3-hydroxylase (*F3H*), flavonoid 3′-hydroxylase (*F3′H*), flavonol synthase (*FLS*), dihydroflavonol 4-reductase (*DFR*), anthocyanidin synthase (*ANS*), and anthocyanidin reductase (*ANR*). The expression of each target mRNA transcript was compared to that of the reference actin gene. Log2 Ratio (relative gene expression) was computed as the ratio of treatment to control gene expression.

**Table 2:** Primers for internal standard gene and genes used in real time PCR.

Gene symbol	Gene name		Primer sequence (5’ - 3’)	Product length (bp)
*ACT*	Actin	Forward Reverse	TGGTAGGTATGGGCCAGAAA GTCATCCCAGTTGCTCACAA	113
*PAL*	Phenylalanin ammonia-lyase	Forward Reverse	AAGGGAAGCCGGAGTTTAC GGAAACGTCGATCAATGG	286
*C4H*	Cinnamic acid 4-hydroxylase	Forward Reverse	GGAGTCGATTGGCACAGAGCT GGCGCATTTCAGTTGATTGTTC	198
*CHS*	Chalcone synthase	Forward Reverse	GGAGGTGGGGCTAACTTTTC GAGCTCCACCTGGTCCAATA	169
*F3H*	Flavanone 3-hydroxylase	Forward Reverse	CTACTCAAGGTGGCCCGATA AATGTGAGATCGGGTTGAGG	210
*FLS*	Flavonol synthase	Forward Reverse	GGTTTGGCCACCTTCTGCTAAG TCTTCACCACCCAACCCT	182
*DFR*	Dihydroflavonol 4-reductase	Forward Reverse	GACAGTGAACGTGCACGGAA CCTTTGTTGCTTCAAATGCTGCT	152
*UFGT*	Flavonoid 3-O- glucosyltransferase	Forward Reverse	AAGAGACCAGAACCCCGTTT AGCTCCAATGCTCTCCGATA	203

### Statistical analysis

Statistical analyses were performed using SPSS software (version 24; IBM Corp., Armonk, NY, USA). To evaluate the effect of two ozone concentrations (100 and 200 ppb) and exposure time (0, 0.25, 0.5, 1, 2, 5, 8, 12, 16, 24, 48, and 72 hours), a one-way analysis of variance (ANOVA) was conducted to compare differences in variance between the treatment. Data shown in all figures are means ± standard error (± SE) (biological experimental replicates; n = 4 for each parameter), *p* < 0.05 was considered as statistically significant (T-test).

## Results

### Growth and morphology of red leaf lettuce

**Figure 2:**
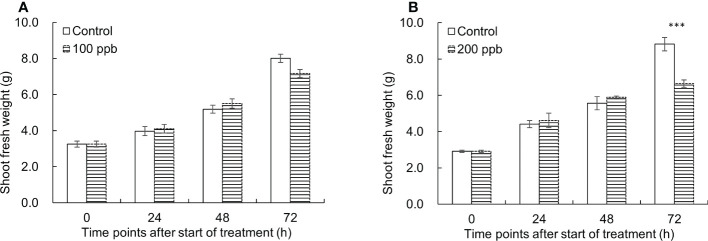
Changes of shoot fresh weight of the control and 100 ppb **(A)** and 200 ppb **(B)** ozone treatments used in this experiment. The vertical vars indicate SE (n=4). Statistically significant differences are indicated at ***p < 0.001 by T-test.

**Figure 2** shows the shoot fresh weight differences between ozone treated and control lettuce plants over time. The growth of the ozone treated-red lettuce seedlings at 100 ppb was almost the same as the control until 48 h of exposure and almost negligible at 72 h. At 200 ppb ozone exposure, the shoot fresh weight was significantly decreased by 1.3 times compared to that of the control at 72 h of treatment.

**Figure 3:**
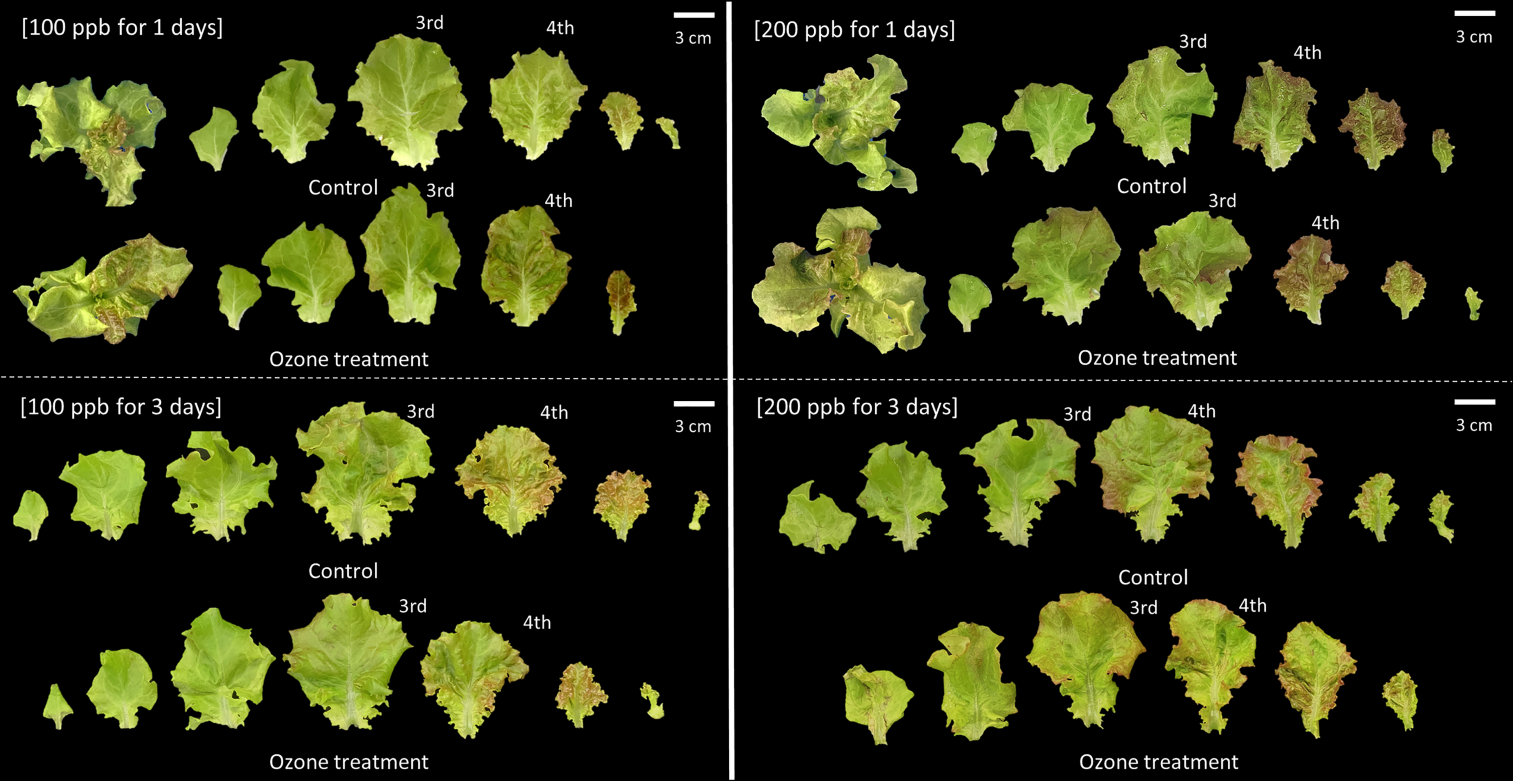
Red leaf lettuce plants exposed to 100 and 200 ppb ozone for 72 h.

Plants were evaluated visually for their appearance, and the size of the ozone-treated plants at 100 and 200 ppb did not differ significantly in comparison to the size of the control plants until 24 h after ozone exposure (**Figure 3**). In comparison to other treatments (control and 100 ppb concentration) lettuce leaves exposed to 200 ppb turned visibly red 24 hours after exposure 24 hours after ozone exposure. The leaves of lettuce exposed to 200 ppb treatment turned red at 24 h compared to those of other treatments (control and 100 ppb concentration).

Plants were visually assessed for appearance and severity, and when plants were exposed to 100 ppb ozone for 72 hours, there was no discernible difference in appearance compared to the control (**Figure 3**). The size of the plants exposed to the 200-ppb ozone treatment appeared smaller.

### Gene expression of phenylpropanoid and flavonoid biosynthetic pathway

**Figure 4:**
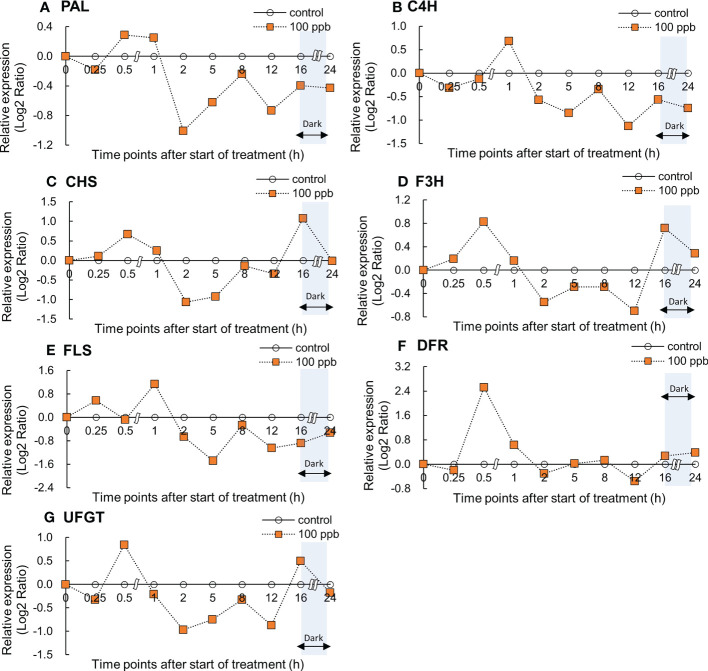
Effect of 100-ppb ozone exposure on relative gene expression (Log2 Ratio) of *PAL* **(A)**, *C4H* **(B)**, *CHS* **(C)**, *F3H* **(D)**, *FLS* **(E)** and *DFR* **(F)**, and *UFGT* **(G)** mRNA in fourth leaf of lettuce plant. The vertical vars indicate SE (n=4). The line graphs indicate Log2 fold change (treatment/control) levels of gene expression.

**Figure 5:**
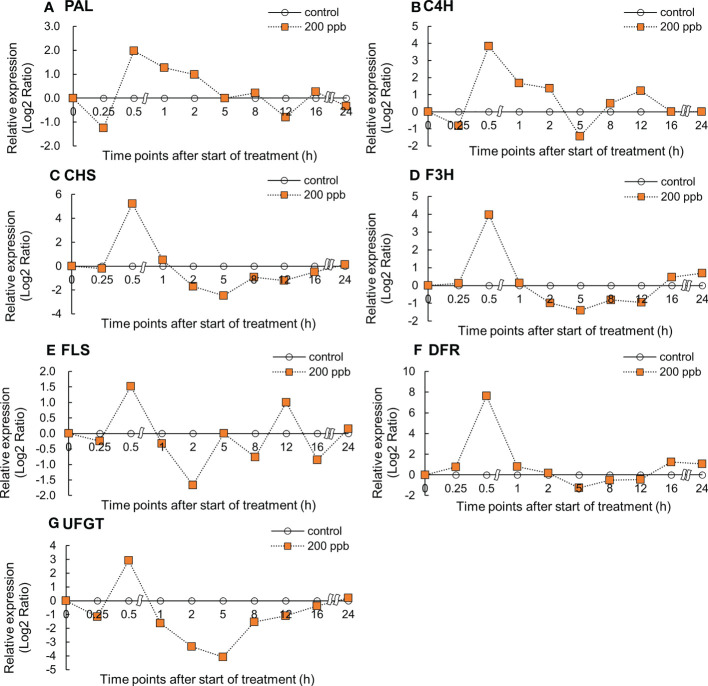
Effect of 200-ppb ozone exposure on relative gene expression (Log2 Ratio) of *PAL* **(A)**, *C4H* **(B)**, *CHS* **(C)**, *F3H* **(D)**, *FLS* **(E)** and *DFR* **(F)**, and *UFGT* **(G)** mRNA in fourth leaf of lettuce plant. The vertical vars indicate SE (n=4). The line graphs indicate Log2 fold change (treatment/control) levels of gene expression.

Relative gene expression in the fourth leaf of red lettuce exposed to both ozone concentrations (100 and 200 ppb) showed an increasing trend with ozone exposure time (**Figures 4**, **5**). The *PAL* and *C4H* relative expression levels reached maxima after 0.5 and 1 h of 100 ppb ozone exposure (**Figure 4**). *PAL* and *C4H* encode the first and second essential enzymes of the phenylpropanoid pathway, respectively. *CHS* and *F3H* expression peaked after 0.5 h of ozone exposure and increased again after 16 h of ozone exposure. *FLS* expression levels peaked after 1 h and decreased thereafter. *UFGT* expression reached a maximum at 0.5 h of ozone exposure and increased once again after 16 h of exposure.

In response to a 200 ppb concentration, relative gene expression exhibited an upward trend compared to 100 ppb (**Figure 5**). All gene expression levels reached maxima at 0.5 h of ozone exposure and decreased thereafter. The *C4H*, *F3H*, *FLS*, *DFR*, and *UFGT* genes showed an increase again between 12 and 24 h of ozone exposure.

### Total phenolics, antioxidant capacity, anthocyanin, and total flavonoid

**Figure 6:**
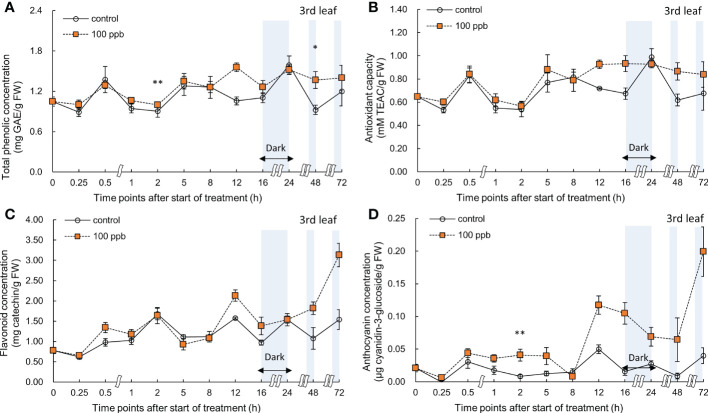
Total phenolic concentration **(A)**, antioxidant capacity **(B)**, total flavonoid concentration **(C)**, and anthocyanin concentration **(D)** of third red leaf lettuce subjected to 100−ppb ozone treatment for 72 h. The vertical vars indicate SE (n=4). Statistically significant differences are indicated at *p < 0.05 and **p < 0.01 by T-test.

**Figure 7:**
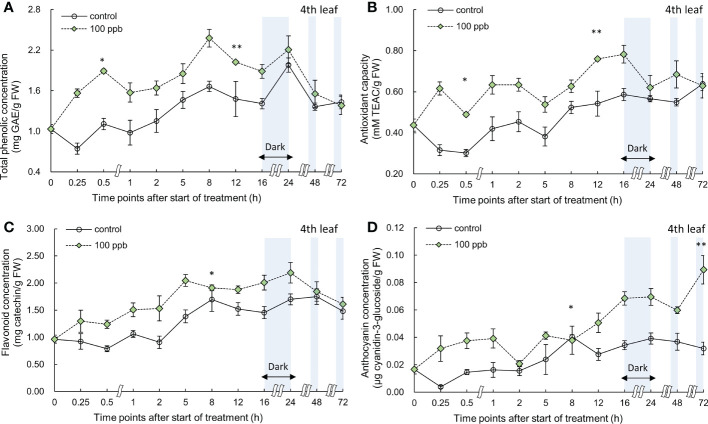
Total phenolic concentration **(A)**, antioxidant capacity **(B)**, total flavonoid concentration **(C)**, and anthocyanin concentration **(D)** of fourth red leaf lettuce subjected to 100−ppb ozone treatment for 72 h. The vertical vars indicate SE (n=4). Statistically significant differences are indicated at *p < 0.05 and **p < 0.01 by T-test.

The concentration of bioactive compounds (total phenolics, anthocyanin, and flavonoids) and the antioxidant capacity in the third and fourth leaves of the red lettuce plants exposed to 100 ppb of ozone were also determined at different times (**Figures 6**, **7**).

A significant increase was observed in the total phenolic concentration of the third leaf compared to that of the control at 2 and 48 h of exposure to 100 ppb of ozone (**Figure 6**). No significant increase was observed in antioxidant capacity and flavonoid concentrations. The anthocyanin concentration of the third leaf showed a significant increase after 2 h of ozone exposure, and a marked increase was observed after 12 h of treatment.

The total phenolics, flavonoids, and anthocyanin concentrations and antioxidant capacity of the fourth leaf exposed to 100 ppb showed an increase immediately after ozone exposure (at 0.25 h) (**Figure 7**). A significant increase was observed in the total phenolic concentration after 0.5 h of treatment, after which it continued to show a higher value than the control. Thereafter, a significant increase was observed at 12 h of ozone exposure. The antioxidant capacity also showed similar results to the total phenolic concentrations. The flavonoid concentration showed a significant increase after 8 h of exposure but showed a tendency to decrease over time. As the ozone exposure time increased, the anthocyanin concentration in lettuce plants exposed to 100 ppb ozone continued to increase. In particular, at 72 h of exposure, anthocyanin concentration significantly increased by about 2.8-fold when compared to that of the control.

**Figure 8:**
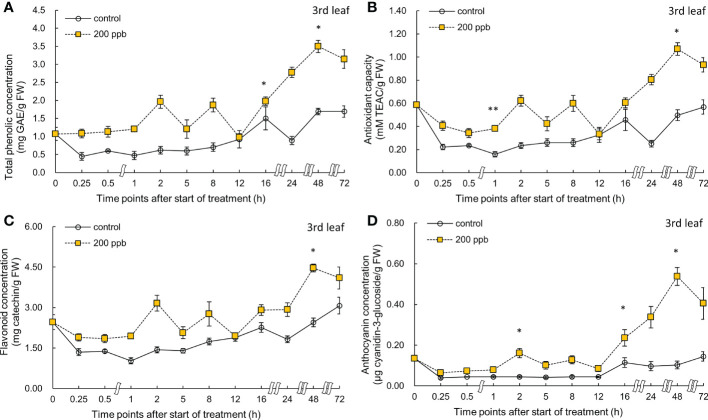
Total phenolic concentration **(A)**, antioxidant capacity **(B)**, total flavonoid concentration **(C)**, and anthocyanin concentration **(D)** of third red leaf lettuce subjected to 200-ppb ozone treatment for 72 h. The vertical vars indicate SE (n=4). Statistically significant differences are indicated at *p < 0.05 and **p < 0.01 by T-test.

**Figure 9:**
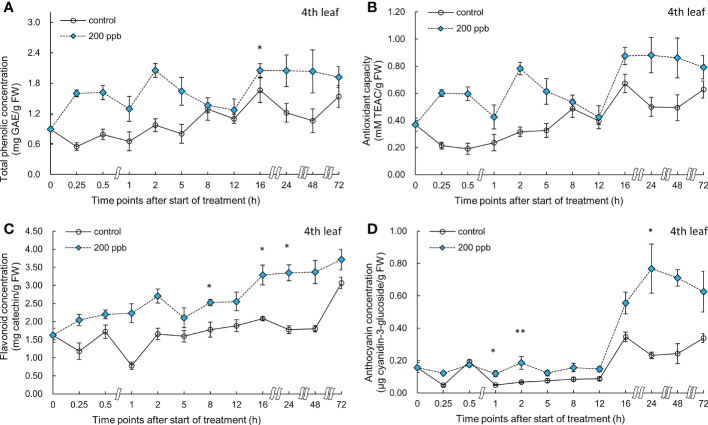
Total phenolic concentration **(A)**, antioxidant capacity **(B)**, total flavonoid concentration **(C)**, and anthocyanin concentration **(D)** of fourth red leaf lettuce subjected to 200-ppb ozone treatment for 72 h. The vertical vars indicate SE (n=4). Statistically significant differences are indicated at *p < 0.05 and **p < 0.01 by T-test.

The bioactive compound concentrations (total phenolic, anthocyanin, and flavonoid) and antioxidant capacity in the third and fourth leaves of the red lettuce plants exposed to 200 ppb of ozone were also determined at different intervals (**Figures 8**, **9**).

Right after exposure to 200 ppb ozone, the concentrations of total phenolics, flavonoids, and anthocyanins and antioxidant capacity in the third leaf were slightly higher than those of the control (**Figure 8**). Bioactive compounds as well as antioxidant capacity exhibited an increasing tendency over time until 48 h. There was a slight decrease after 72 h of exposure to ozone. At 48 h of exposure, the total phenolics, antioxidant capacity, flavonoids, and anthocyanin concentrations were all significantly higher than those in the control by 2.1- 1.07-, 1.83-, and 5.23-fold, respectively.

Bioactive compound concentrations in the fourth leaf exposed to 200 ppb of ozone were also high compared to those of the control (**Figure 9**). Total phenolic concentration was significantly increased after 16 h of exposure. Flavonoid concentration was significantly increased at 8, 16, and 24 h of ozone exposure (1.42-, 1.58-, and 1.88-times). Anthocyanin concentration started to increase after 1 h; the highest value was obtained at 24 h after exposure to ozone (3.31-times) compared to the control.

### Hydrogen peroxide

**Figure 10:**
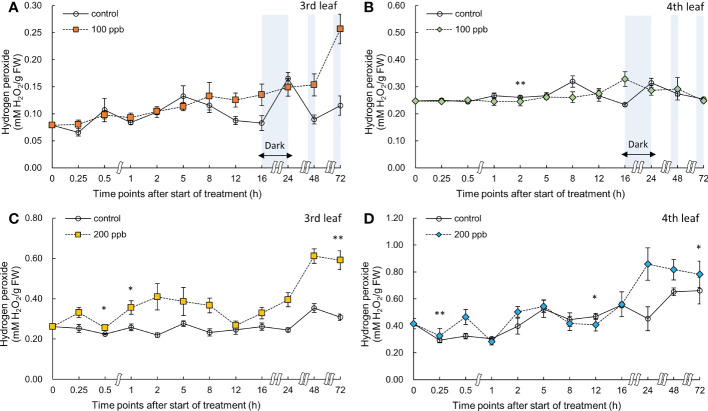
Hydrogen peroxide of third **(A, C)** and fourth **(B, D)** red leaf lettuce subjected to 100- and 200-ppb ozone treatment for 72 h. The vertical vars indicate SE (n=4). Statistically significant differences are indicated at *p < 0.05 and **p < 0.01 by T-test.

Hydrogen peroxide accumulation in red leaf lettuce varied depending on the ozone concentration and the order of the exposed leaves (**Figure 10**). The H_2_O_2_ content of lettuce leaves exposed to 100 ppb of ozone did not show any increase in both the third and fourth leaves. However, a significant increase was observed in the H_2_O_2_ content of the third leaf exposed to 200 ppb ozone after 0.5 h of ozone exposure compared to the control. The H_2_O_2_ content continued to show a higher value than the control; the highest value was obtained at 72 h of exposure (1.73-fold). The fourth leaf also showed a significant increase in H_2_O_2_ content in the leaves exposed to 200 ppb ozone after 0.25 h and showed a 1.11-times higher value compared to that of the control at 72 h of exposure.

## Discussion

### Effect of ozone concentration on growth

Both 100 and 200 ppb ozone treatments did not show a significant effect on growth until 48 h of ozone exposure (**Figures 2**, **3**). As the ozone exposure continued for up to 72 h, there was a significant reduction in the growth of the ozonated seedlings. In particular, 200 ppb treatment significantly decreased the shoot fresh weight of red leaf lettuce plants. Damages caused by ozone exposure to plants depend on the ozone regime and plant species (Ferdinand et al., 2000; Singh et al., 2018). However, under conditions of chronic ozone exposure, increasing oxidative stress would cause damage to plant growth and quality (Khaling et al., 2015; Pandey et al., 2018). During respiration, the leaf absorbs ozone through its stomata; this ozone can alter the chemical composition of cells as a whole (Lee, 1968). As ozone reacts, there is an instantaneous increase in the activity of reactive oxygen species (ROS) (Mehlhorn et al., 1990; Buchanan-Wollaston and Morris, 2000; Booker et al., 2009). Many studies have reported the effects of ozone stress-induced ROS on plants (Podila et al., 2001; De Temmerman et al., 2002).

In other studies, ozone has significantly reduced the overall development, leaf area, and biomass (yield) of most plants (Oksanen et al., 2001; Menéndez et al., 2017; Singh et al., 2018). However, some plants exhibit either negative or positive photopigment responses to extreme ozone stress (Pääkkönen et al., 1998a; Woo et al., 2004; Singh et al., 2009; Singh, 2017). The critical ozone concentration may vary depending on the dosage of ozone exposure, environmental conditions, leaf thickness, leaf age, leaf development stages, and plant varieties or cultivars (Karlsson et al., 1995; Calatayud et al., 2007; Sarkar and Agrawal, 2012) In the present study, no significant changes were observed in the fresh weight of red leaf lettuce after 3 days of exposure to 100 ppb ozone, but a significant decrease was observed in the 200 ppb ozonated plants compared with the control. These results suggest that a dose of approximately 200 ppb × 48 h is a critical dose in the red leaf lettuce cultivar and experimental conditions used in this study. It has been reported that due to a reduction in photosynthetic capacity, carbon assimilation and a reduced leaf lifetime, ozone-induced senescence acceleration contributes to economic yield losses of plants (Ainsworth, 2017; Emberson et al., 2018). The ROS produced in the plant by ozone concentration of 200 ppb for 3 days probably decreased the photosynthetic capacity of lettuce, thereby inhibiting its growth.

In a previous study, ozone exposure of 150 ppb for 1 h had no effect on dry matter growth of Pak-Choi plants, whereas 4 h of exposure resulted in growth reduction (Han et al., 2020). Seventy ppb ozone for 2 days had no effect on growth parameters such as leaf area and reproductive development, whereas 10 d ozone exposure reduced the whole growth of *Brassica campestris* plants (Black et al., 2007). When plants are exposed to extreme levels of ozone, injury such as tissue damage (cell necrosis), chlorosis and stomata closure occur due to excessive accumulation of ROS (Jacobson and Hill, 1970; Heath, 2008). Moeder et al. (2002) demonstrated that the regions/tissue where hydrogen peroxide is produced and chlorosis occurs are similar. However, in our study no tissue injury was observed in lettuce leaves even after exposure to 200 ppb for 3 days. These results indicate that the hydrogen peroxide generated during the 3 days was not at a level that would cause serious damage to the leaves.

### Gene expression of phenylpropanoid and flavonoid pathways

Immediately after ozone exposure, the expression of genes involved in the phenylpropanoid and flavonoid biosynthetic pathways in red leaf lettuce plants increased in both 100 and 200 ppb ozone treatments (**Figures 4**, **5**). Except for the *C4H* and *FLS* genes, gene expression in lettuce plants exposed to 100 ppb ozone reached a peak after 0.5 h of ozone exposure. However, the expression of all genes in lettuce plants exposed to 200 ppb of ozone increased rapidly after 0.5 h and showed a tendency to decrease after reaching a peak (**Figure 5**). Plants respond to ozone stress by inducing a variety of defense responses. Active defense mechanisms include an increase in phenylpropanoid pathway gene transcripts, such as those encoding *PAL* and *CHS* (Eckey-Kaltenbach et al., 1997; Pääkkönen et al., 1998b). When plants are exposed to low levels of ozone, physiological and metabolic changes are observed. With the exception of serious injuries such as tissue injury or stomata closure, the great majority of these disruptive changes generally begin at the level of gene expression (Heath, 2008). Previous studies found that the increase in the gene expression of secondary metabolites in plants exposed to ozone followed different patterns depending on the type of crops (species or cultivar), ozone concentration, and exposure time. *PAL* gene expression in *Salvia officinalis* plants exposed to 120 ppb (5 h day^−1^) significantly increased after 5 h of ozone exposure (Marchica et al., 2020); *PAL* mRNA in *Arabidopsis* leaves exposed to 300 ppb of ozone dramatically increased after 3 h (Sharma et al., 1996). According to Tuomainen et al. (1996), when birch plants were exposed to 150 ppb ozone for 96 h (8 h of pulse treatment), *PAL* was rapidly but transiently induced after 8 h of ozone exposure. *PAL* is an upstream gene that directs phenylpropanoid metabolism to its primary branch (**Supplementary Figure S2**). These results indicate that the expression of many genes encoding secondary metabolite pathways increased rapidly within 1 h of ozone exposure.

The phenylpropanoid and flavonoid biosynthetic pathways are certainly the most important pathways for the synthesis of antioxidant phytochemical compounds in plants, such as phenols, flavonoids, and lignin (Guidi et al., 2005). In our study, the expression of genes related to phenylpropanoid and flavonoid biosynthetic pathways increased immediately after ozone exposure (**Figures 4**, **5**). It is possible that secondary metabolite compounds such as total phenolics, antioxidant capacity, flavonoids, and anthocyanins were increased. In several ozonated plants, an increase in the expression of genes related to secondary metabolites has been linked to an increase in the production of bioactive compounds (Eckey-Kaltenbach et al., 1994; Booker and Miller, 1998; Cabané et al., 2004).

### Changes in bioactive compounds in relation to ozone concentrations according to leaf age

The concentration of bioactive compounds in red leaf lettuce leaves exposed to ozone showed different responses according to the ozone concentration (**Figures 6**–**9**). The total phenolic concentration, antioxidant capacity, and total flavonoid concentration of third leaves exposed to ozone did not show a significant difference in the 100-ppb ozone treatment compared to that of the control. The fourth leaf treated with 100 ppb ozone exhibited a relatively greater increase than the third leaf. However, in the case of lettuce leaves exposed to 200 ppb ozone, the bioactive compounds, except for anthocyanins, showed a tendency to increase rapidly immediately after ozone exposure regardless of leaf position (third or fourth). Thus, the ozone concentration affected the accumulation of bioactive compounds in red leaf lettuce. Antioxidant enzymes such as APX and CAT in wheat plants of the jointing stage exposed to various ozone concentrations showed increased activities at 40 ppb ozone treatment and decreased in other treatments (Liu et al., 2015). In addition, the concentrations of ascorbate and glutathione were significantly higher at 40 ppb than at 80 ppb in two soybean plants with different tolerances to ozone (Tiwari and Agrawal, 2011). Thus, there is an effective ozone concentration for the accumulation of antioxidants in different plants.

To confirm the effect of the light period, an experiment of 100 ppb of ozone concentration was performed with 16 h light period (**Figures 6**, **7**). During the 8 h dark period, the plant failed to photosynthesize and did not accumulate new carbohydrates or precursors for the synthesis of secondary metabolite compounds, including antioxidants. Therefore, 100 ppb ozone treatment with an 8 h dark period may have a smaller positive effect on the accumulation of bioactive compounds than 200 ppb with continuous lighting. In addition, there was a significant reduction in the ascorbic acid content in both varieties of lettuce (*Lactuca sativa* and *Lactuca serriola*) which were exposed to ozone under dark conditions (Goumenaki et al., 2021). However, when the plants were transferred to the light conditions, the ascorbic acid content increased rapidly, showing twice the concentration of that under the dark condition (Goumenaki et al., 2021). These results indicate that plants are substantially more impacted by equivalent ozone flux absorbed at night than during the day, and that the detoxification potential of plants is compromised at night time (Wieser and Havranek, 1995; Lloyd et al., 2018). In addition, the nighttime reduction of ascorbate content and/or redox homeostasis may increase the sensitivity of the plants to ozone (Maddison et al., 2002; Sanmartin et al., 2003; Höller et al., 2015). For this reason, it is thought that the accumulation of bioactive compounds under 100 ppb ozone with a light period did not increase when compared to that treated with 200 ppb ozone (**Figures 6**–**9**). In addition, in the case of a control plant treated with a photoperiod of 16 hours in a 100 ppb treatment, it was observed that the total phenolic concentration, antioxidant capacity, and flavonoid concentration were increased at 24 hours after dark period compared to 16 hours after light period (**Figures 6**–**7**). This increase may be the result of daily rhythms of phytochemicals. Previous studies have shown that photosynthesis, carbohydrate, and amino acid levels can depend on circadian clock patterns (Vazirifar et al., 2021; Francisco and Rodriguez, 2021). Vazirifar et al. (2021) reported that the photoperiod (light/dark period) controls the carbon and energy flows involved in the production and consumption of starch and the activation of phenylalanine-related enzymes. Typically, at the end of the dark period/or at the beginning of the light period, phenolic compounds exhibiting antioxidant properties are accumulated higher in plants (Hasperué et al., 2011; Soengas et al., 2018). The polyphenols in plants are raised to protect them from damage caused by too much light, and plants adjust their biological clocks based on the daily cycle (Francisco and Rodriguez, 2021). It is also possible that the antioxidant accumulation pattern of plants may have changed due to the combined action of the circadian rhythm of phytochemicals and ozone concentration according to the photoperiod. The increasing pattern of bioactive compounds was different according to the leaf age, even with the same ozone concentration (**Figures 6**–**9**). A higher accumulation was observed at 200 ppb concentration in the third leaf (older) than in the fourth leaf (younger). Therefore, the response to ozone exposure can vary greatly with leaf position. However, the opposite has been reported in soybean plants exposed to ozone (70 ppb for 5 days). In soybeans, older leaves are first exposed to stressors than younger leaves (Bailey et al., 2019). While considering the effects of ozone in plants, these observations emphasize the importance of understanding the age of the leaves. Leaf age could be related to the ability to modulate stomatal conductance, oxidative signaling, and activation of defense mechanisms (Vingarzan, 2004; Mill et al., 2011; Schneider et al., 2017). However, studies on the effect of ozone on the accumulation of bioactive compounds in different-aged leaves remain limited.

The ozone concentration of 200 ppb may have acted as a strong stressor on the third and fourth leaves of lettuce (**Figure 9**). The H_2_O_2_ concentration in the third leaf was approximately 1.9-fold higher than that in the control at 72 h of ozone exposure. However, the concentration in the fourth leaf was approximately 1.3-fold higher than that in the control. These results suggest that a constant 200 ppb ozone concentration can have a different effect on the accumulation of bioactive compounds according to leaf age. The ozone concentration of 100 ppb in the third leaf showed no significant difference compared to the control in the total phenolic concentration, flavonoid concentrations, and antioxidant capacity during all of the treatment periods. However, in the fourth leaf, a significant increase was observed in the antioxidant capacity compared to the control at 0.5 and 12 h of 100 ppb ozone exposure (**Figure 7**). These results indicate that the 100-ppb concentration in the fourth leaf can also help bioactive compound accumulation. The total phenolics, antioxidant capacity, flavonoids, and anthocyanin concentrations of the third leaf exposed to 200 ppb ozone continued to increase until 48 h and then decreased after 72 h of ozone exposure. In previous studies, researchers have also found that bioactive compounds increase and then decrease up to a certain treatment level (Taulavuori et al., 2016; Cammarisano et al., 2020). Thus, under high levels of ozone, the third leaf of lettuce plants gradually lose the ability to activate secondary metabolism. This time-dependent decrease in the accumulation of antioxidant properties was also observed in *Salvia officinalis* exposed to 120 ppb ozone (5 h day^−1^) (Marchica et al., 2021).

### Relationship between ROS and bioactive compounds in red leaf lettuce plants

The phytotoxic effects of ozone are mostly attributable to the ozone-induced generation of ROS that exceed the capacity of plants to maintain ROS below the tolerance threshold. Ozone impacts on plants are determined by the balance between ozone uptake and cellular antioxidant potential (Di Baccio et al., 2008). After ozone penetrates the leaf tissue, it interacts with extracellular antioxidants, which appear to stimulate ascorbate synthesis and/or its transport between compartments. The antioxidants in the apoplast will respond to ozone within the cell wall space, thereby protecting the membrane from ozone damage. The generation of H_2_O_2_ near the membrane appears to initiate both a pathogen-like response with an increase in H_2_O_2_ production (Zheng et al., 2000; Conklin and Barth, 2004; Heath, 2007; Heath, 2008). The effects of ozone can be characterized as either acute or chronic, based on the exposure intensity and duration of ozone treatment. Long-term exposure to relatively low ozone concentrations reduces photosynthesis and growth and promotes leaf senescence, but short-term exposure to high ozone concentrations induces leaf injuries reminiscent of the hypersensitive cell death triggered during plant-pathogen interactions (Schlagnhaufer et al., 1995; Mahalingam et al., 2003; Castagna and Ranieri, 2009).

In our study, it is possible that ROS (especially, H_2_O_2_) continued to increase as a result of the plant-pathogen interaction reaction caused by the short-term exposure of high ozone concentrations (**Figure 10**). Ascorbic acid and glutathione in the apoplast are two antioxidants that regulate ROS generated through ozone treatment, and are found in high concentrations in the leaves of all plant species (Iriti and Faoro, 2007; Foyer and Noctor, 2011). Regardless of leaf order, H_2_O_2_ concentration started to increase significantly immediately after 200 ppb ozone exposure (0.25 h) in the present study (**Figure 10**). After ozone enters the plant leaves, it is possible that H_2_O_2_ reacted with apoplastic antioxidants such as ascorbic acid and glutathione. In our results, the bioactive compounds of lettuce accumulated under both ozone treatments (100 and 200 ppb) and showed up-and-down mobility regardless of leaf order. These results may be due to the rapid oxidation-reduction reactions between antioxidants and ROS in cells. As ozone exposure time increased, ROS burst appeared; it is possible that antioxidants were generated as a defense mechanism (Bolwell, 1996). In 200 ppb ozonated third leaf, a rapid increase in the production of secondary metabolites as a defense mechanism in response to ozone entry into leaves was observed after 24 h of exposure.

Under conditions of oxidative stress, the phenolic compounds generated by the phenylpropanoid and flavonoid biosynthetic pathways are strong antioxidants in plant tissues (Taiz and Zeiger, 2002). Anthocyanins, which are flavonoids, are water-soluble pigments that concentrate in the vacuole (Chalker-Scott, 1999). According to the results of previous studies, ozone can stimulate the biosynthesis of anthocyanins in plants. Various stress factors, including water stress (osmotic), UV-irradiation, nutrient deficiency, low temperature, and ozone, strongly promote the production of anthocyanin (Chalker-Scott, 1999). Purified anthocyanin solutions are four times more effective in scavenging ROS than α-tocopherol and ascorbate, indicating that these components have a significant antioxidant capacity (Gould et al., 2000; Feild et al., 2001; Gould, 2004). Thus, the accumulation of anthocyanins may be essential for ozone stress tolerance (or sensitivity), since it may protect ozonated leaves from damage caused by ozone-induced ROS (Bortolin et al., 2014). The anthocyanin content varies among different cultivars or species in vegetable plants exposed to ozone. Broccoli plants exposed to 70 µg m^−3^ of ozone did not show a significant difference in anthocyanin content compared to that of the control, but a significant 2.5-fold increase was observed in Chinese cabbage (Rozpądek et al., 2015). It could be said that the lettuce used in this experiment responded immediately to ozone because it was a red leaf lettuce accumulating anthocyanins as it matures. However, when plants are exposed to high ozone concentrations, ozone may destroy chlorophyll and decompose anthocyanins (Rathore and Chaudhary, 2019). In our results, anthocyanin concentrations decreased from 48 to 72 h of ozone exposure (**Figures 6**–**9**). These results suggest that ozone exposure for longer than 72 h may adversely affect anthocyanin biosynthesis. Three days of ozone exposure with 200 ppb inhibited the fresh weight of lettuce significantly, but did not show the difference at 2 days of ozone exposure (**Figure 2**). A significant accumulation of bioactive compounds both in the third and fourth leaves may have been observed at around 48 h of ozone exposure. Therefore, it can be concluded that 2 days of ozone exposure with 200 ppb can be useful for the production of photochemical-rich leaf lettuce. Our results suggest that ozone control is a novel method as preharvest treatment effective for increasing antioxidant bioactive compounds in lettuce in a PFAL.

## Conclusions

A PFAL, also known as a vertical farm, is an advanced plant production system for the commercial production of leafy vegetables. Ozone concentration can be controlled easily using a simple control apparatus in a PFAL. This study was conducted to confirm the complex effect of ozone on red leaf lettuce. In the case of 100 ppb, the concentrations of bioactive compounds and antioxidant capacity were increased in the relatively young fourth leaf. However, with 200 ppb, those were found to be considerably high in the third leaf. Therefore, the sensitivity of red leaf lettuce plants to ozone-induced oxidative stress is determined by apoplast antioxidants and/or redox homeostasis. Although the degree of accumulation of antioxidant-bioactive compounds may vary between leaf age depending on the ozone concentration, it was confirmed that the concentrations of phytochemicals such as anthocyanins could be effectively increased in a few days. Also, in this study, a complex reaction between photoperiod and ozone was confirmed. These results suggest that if the ozone treatment is applied immediately prior to harvest (e.g., by progressively increasing the ozone concentration for 2 days and combined treatment of photoperiod and ozone), the amount of bioactive compounds can be enhanced without inhibiting growth. Our results suggest that ozone control is a novel method effective for increasing antioxidant bioactive compounds in lettuce in a PFAL or a vertical farm.

## Data availability statement

The original contributions presented in the study are included in the article/**Supplementary Material**. Further inquiries can be directed to the corresponding authors.

## Author contributions

Performance of experiments, sample collection, analyses of chemical data, writing-original draft preparation: J-HL; writing-review and editing, conceptualization, experimental design, supervision and funding acquisition: EG. All authors read and agreed to the final version of the manuscript.

## Funding

This work was supported by the Ministry of Economy, Trade, and Industry of Japan in the form of a grant-in-aid from the project <*Development of Fundamental Technologies for the Production of High-Value Materials Using Transgenic Plants*> and funded by the Program on Open Innovation Platform with Enterprises, Research Institute and Academia, Japan Science and Technology Agency (JST-OPERA, JPMJOP1851).

## Conflict of interest

The authors declare that the research was conducted in the absence of any commercial or financial relationships that could be construed as a potential conflict of interest.

## Publisher’s note

All claims expressed in this article are solely those of the authors and do not necessarily represent those of their affiliated organizations, or those of the publisher, the editors and the reviewers. Any product that may be evaluated in this article, or claim that may be made by its manufacturer, is not guaranteed or endorsed by the publisher.

## Supplementary material

Supplementary Figure 1 The control and different ozone treatments used in this experiment.

Supplementary Figure 2 Phenylpropanoid, flavonoid, and anthocyanin biosynthetic pathways. The genes analyzed in this study are marked in red.
