# Supplementary figures and images for: Ozone control as a novel method to improve health-promoting bioactive compounds in red leaf lettuce (Lactuca sativa L.)
Source: Front Plant Sci. 2022 Dec 5;13:1045239. doi: 10.3389/fpls.2022.1045239 (PMC9760822; doi:10.3389/fpls.2022.1045239)

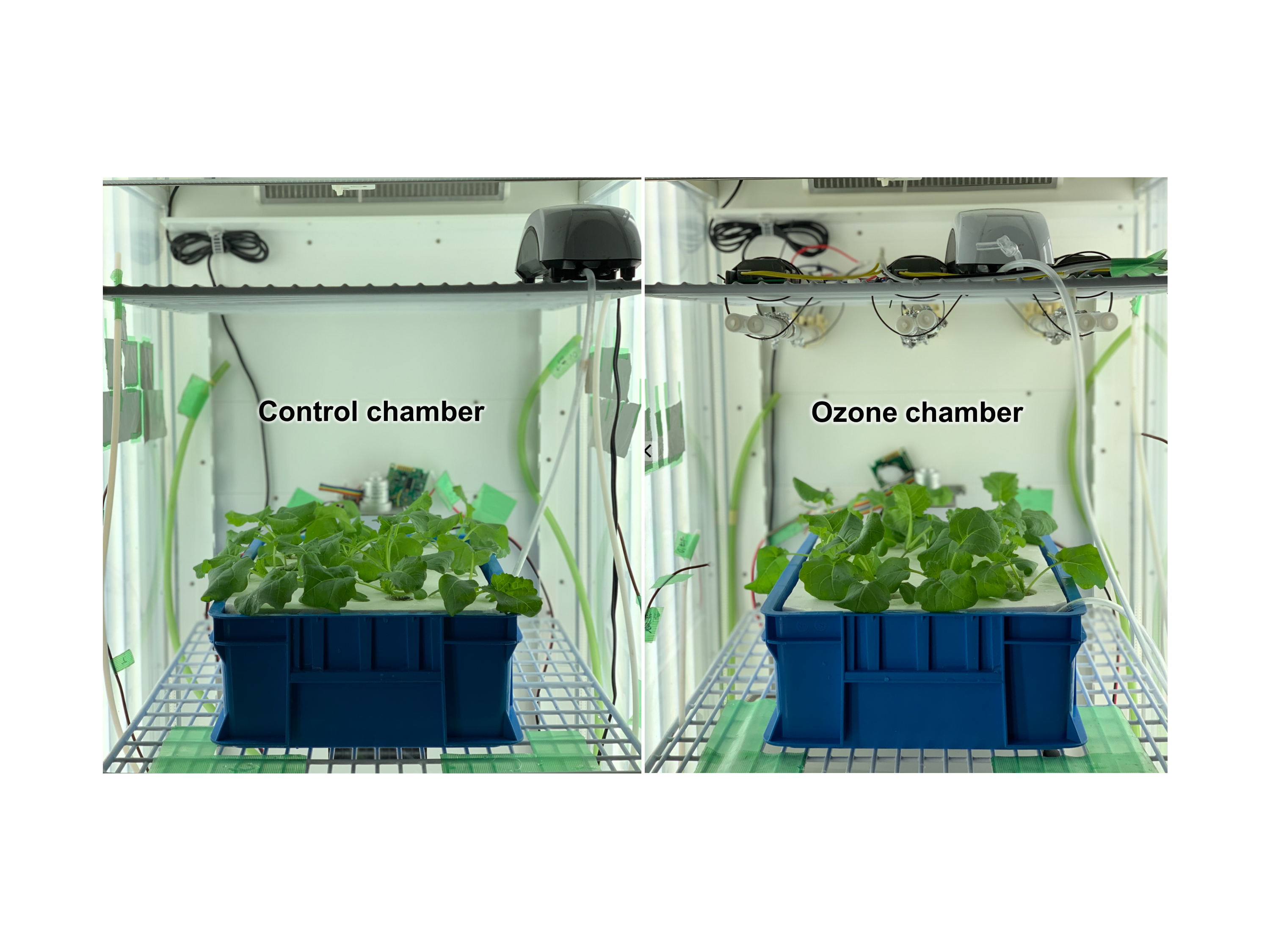

Supplement: Supplementary Figure 1 — The control and different ozone treatments used in this experiment. [file Image_1.tif]

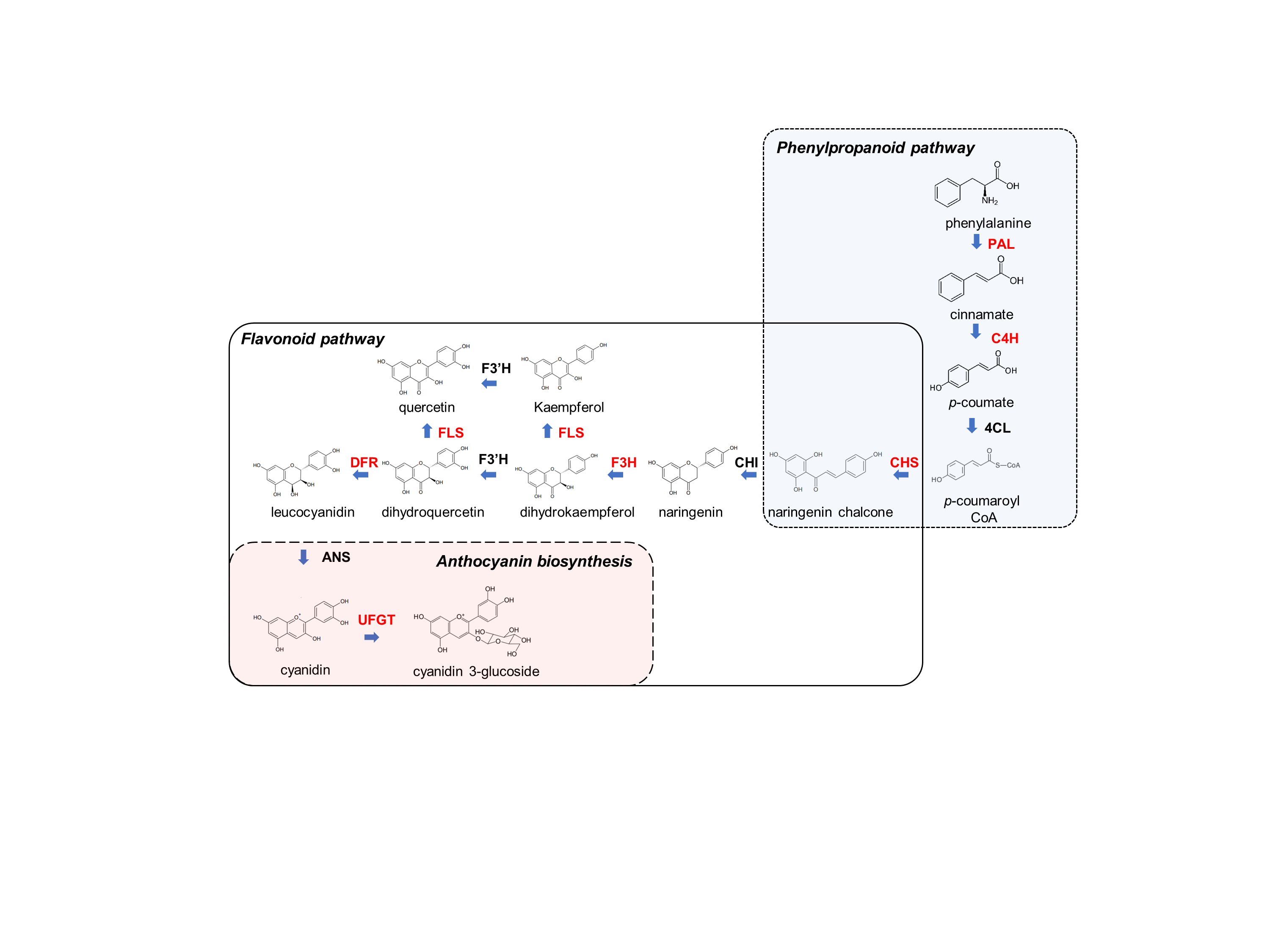

Supplement: Supplementary Figure 2 — Phenylpropanoid, flavonoid, and anthocyanin biosynthetic pathways. The genes analyzed in this study are marked in red. [file Image_2.tif]
